# Inhibition of PRC1 elicits immunogenic cell death by triggering ROS-dependent ER stress in colorectal cancer via the Wnt/*β*-catenin signaling pathway

**DOI:** 10.1186/s13062-025-00685-0

**Published:** 2025-08-22

**Authors:** Wei Wang, Lijiang Zhou, Xinyu Zhang, Zheng Li

**Affiliations:** 1 Department of Integrated Traditional Chinese and Western Medicine, Cancer Hospital of China Medical University, Liaoning Cancer Hospital and Institute, No.44 Xiaoheyan Road, Dadong District, Shenyang, 110042 Liaoning Province People’s Republic of China; 2 Department of Oncology, Affiliated Hospital of Liaoning University of Traditional Chinese Medicine, Shenyang, 110032 People’s Republic of China

**Keywords:** Protein regulator of cytokinesis 1, Colorectal cancer, Immunogenic cell death, PD-L1, Wnt/*β*-catenin

## Abstract

Due to the low response rate and severe side effects, the clinical efficacy of current immunotherapy for patients with colorectal cancer (CRC) remains unsatisfactory. Induction of immunogenic cell death (ICD) has been evidenced to be conducive to enhancing the survival benefit of immune checkpoint blockade (ICB) therapy. Protein regulator of cytokinesis 1 (PRC1) has been proven to be a tumor promoter in CRC and an immune marker. However, whether and how PRC1 is involved in the ICD regulation in CRC remains undiscovered. The current study identified the upregulation of PRC1 in CRC tissues and its prognostic value via bioinformatics analyses. Similarly, we determined the close correlation between PRC1 and ICD. In addition, knockdown of PRC1 induced ICD and downregulated PD-L1 expression in CRC cells, which was attenuated by ER stress inhibitor 4-PBA. PRC1 silencing elicited ER stress, but this effect was partially rescued by the ROS scavenger N-acetylcysteine. Mechanism investigation revealed that PRC1 could stimulate Wnt/*β*-catenin activation in CRC cells. According to results of rescue assays, activation of Wnt/*β*-catenin by BML-284 could partially reverse the effects of PRC1 knockdown on ER stress and ICD in CRC cells. Finally, the in vivo experiments demonstrated that silencing of PRC1 restrained tumor growth in CRC animal models. In conclusion, this study verified that inhibition of PRC1 expression could induce ICD in CRC by triggering ER stress via the Wnt/*β*-catenin signaling pathway. These findings highlight a novel molecular pathway whereby PRC1 exerts carcinogenic role in tumor immune microenvironment through ICD in CRC.

**Supplementary Information:**

The online version contains supplementary material available at 10.1186/s13062-025-00685-0.

## Introduction

Colorectal cancer (CRC) ranks fourth among all highly prevalent cancer worldwide, and its pathogenesis is complicated. According to epidemiological studies, inflammatory bowel disease, lifestyle, environmental changes, and genetic alterations are all causes of CRC [1]. Immunotherapy has been applied for CRC treatment, with median overall survival rate of 66% at 24 months [2]. Currently, inhibition of PD-1-PD-L1 axis and other immune regulatory checkpoints is at the forefront of immunotherapy for various human cancers [3]. Immune checkpoint blockade (ICB) therapy such as PD-1-PD-L1 blockade has shown efficacy in microsatellite instability (MSI) CRC, which can produce more neoantigens, thereby increasing the likelihood of tumor immune recognition [2]. However, tumors that are devoid of T cells and antigens are usually failed to respond to anti-PD1 [4]. Therefore, the way to improve the clinical efficacy of ICB therapy is to create an immunogenic environment within immunocompromised or immunosuppressed tumors.

Immunogenic cell death (ICD) makes the immune system recognize tumor cells and transform tumors into ‘in-situ vaccines’ [5, 6]. Agents that induce ICD have been used to shape tumor immunogenicity [7, 8]. An important characteristic of ICD is the release of damage-associated molecular patterns (DAMPs), including chaperone calreticulin (CALR or CRT) [9], the release of ATP, and high-mobility group box 1 (HMGB1) [10]. These DAMPs acting as danger signals can be recognized by the innate immune system so as to initiate a strong anti-tumor response [11]. Considering these immunostimulatory properties, ICD inducers have been thought to be useful in combination immunotherapy to increase tumor sensitivity to ICD therapy [12]. The current study sought to uncover potential therapeutic targets that could be used to induce ICD in CRC. Protein regulator of cytokinesis 1 (PRC1) is a member of microtubule-associated proteins (MAPs) family, with location at human chromosome 15 [13]. PRC1 has been proven to be a tumor promoter in CRC [14] and an immune cell marker [15]. Moreover, PRC1 is associated with immunotherapy [16, 17] and immune suppression [18]. Growing evidence reveals that PRC1 presents ectopic expression in some malignant tumors and involves in malignant progression of human cancers [19, 20]. In light of these previous findings, we hypothesize that PRC1 may be involved in the regulation of immune surveillance and ICD during the development of CRC.

Endoplasmic reticulum (ER) stress determines the immunogenicity of cell death within tumors [21], which can recruit antigen-presenting cells (APCs) to recognize and engulf dying tumor cells by releasing DAMPs as “come and eat me” signals. Subsequently, APCs can activate T cells to attack tumor by displaying tumor peptides. ER stress-induced apoptosis has similar characteristics with ICD and can trigger a strong immune response to dead-cell antigens [6]. However, whether PRC1 is involved in the development of CRC through the ER pathway remains unknown. The current study focused on exploring the role of PRC1 in modulating ER stress-dependent ICD in CRC progression and investigating the specific mechanism.

Wnt/*β*-catenin is a signal transduction pathway with high conservation, which is one of the most representative cancer-related signaling pathways [22]. According to previous reports, Wnt signaling pathway can affect CRC development [23] and immune surveillance [24]. Importantly, targeting Wnt-signaling pathway can enhance the efficacy of cancer immunotherapy [25]. Additionally, inhibition of Wnt/*β*-catenin can promote ICD and inhibit conjunctival melanoma progression [26]. However, it remains unknown whether PRC1 can affect ICD in CRC via altering the activation of Wnt/*β*-catenin. This study aims to explore whether the Wnt/*β*-catenin signaling pathway acts as a downstream effector to coordinate the carcinogenic function of PRC1 in CRC.

## Materials and methods

### Data collection and analysis

Top 30 genes significantly upregulated (adjp < 0.05, logFC > = 1) in 30 tumor tissues obtained from CRC patients and 34 tumor tissues collected from MSI CRC patients were downloaded from Gene Expression Omnibus (GEO) database (https://www.ncbi.nlm.nih.gov/geo/, accession numbers: GSE74602 and GSE24514). A total of 15,542 genes differentially expressed in CRC tissues were found out from GEO database by analyzing two GEO datasets (GSE74602 and GSE24514). The expression profile data of PRC1 in 619 tumor tissues obtained from COAD and READ patients was downloaded from The Cancer Genome Atlas (TCGA) database (https://tcga-data.nci.nih.gov/tcga). The expression data of PRC1 in 51 normal colon tissues was obtained from the Genotype-Tissue Expression (GTEx) database (https://gtexportal.org/). The expression correlation between PRC1 and three ICD markers (CD247, CRT, and HMGB1) in tumor tissues of COAD and READ patients was analyzed and downloaded from GEPIA (http://gepia2.cancer-pku.cn/#analysis). A total of 1811 protein coding genes associated with ICD were screened out from Genecards (https://www.genecards.org/). IHC data of PRC1 expression in tumor and normal tissues collected from COAD and READA patients were obtained from HPA database (https://www.proteinatlas.org/). PRC1 expression in patients with ICB therapy was downloaded from ICBatlas database (https://guolab.wchscu.cn/ICBatlas/#!/). PRC1-related genes were screened out through Pan-cancer analysis of whole genomes (*P* < 0.05) in cBioPortal (http://www.cbioportal.org/).

### Gene set enrichment analysis (GSEA)

To detect the downstream pathway of PRC1, KEGG pathway enrichment analysis was designed and conducted. PRC1-related differentially expressed genes were enriched and analyzed in hallmark gene sets using GSEA, and the results were visualized.

### Cell culture and chemical treatment

CRC cells (LoVo, Caco-2, and SW480) were purchased from the Cell Bank of Chinese Academy of Sciences (Shanghai, China). LoVo cells were cultured in F-12 K medium (Gibco) with additional 10% fetal bovine serum (FBS). Caco-2 cells were cultured in minimum essential medium (Gibco) with additional 1% non-essential amino acids and 20% FBS. SW480 cell line was cultured in Leibovitz’s L-15 medium (Gibco) with additional 10% FBS. The normal colon epithelial cell line (FHC) obtained from American Type Culture Collection (Manassas, VA, USA) was cultured in a mixed medium containing Ham F-12 medium and DMEM (1: 1) with supplementation of 25 mM HEPES, 10 ng/ml cholera toxin, 5 µg/ml insulin, 5 µg/ml transferrin, 100 ng/ml hydrocortisone, 20 ng/mL human recombinant EGF, and 10% FBS. All cell lines were incubated at 37 °C in a humid atmosphere containing 5% CO_2_.

To confirm whether ROS was involved in the regulatory effect of PRC1 on ER stress, CRC cells were pretreated with the ROS scavenger N-acetylcysteine (NAC; 2 mM, MCE, Shanghai, China) for 2 h prior to gene transfection to block ROS generation. To determine the involvement of ER stress in PRC1-mediated ICD, CRC cells were treated with the ER stress inhibitor 4-PBA (4 mM, MCE) for 48 h. For activation of the Wnt/*β*-catenin signaling pathway, CRC cells were treated with a Wnt activator BML-284 (0.5 µM, MCE) for 48 h.

### Cell transfection

Small interfering RNA (siRNA) targeting PRC1 (si-PRC1) and its negative control (si-con) was designed and synthesized by Guangzhou Ribobio (China). Lipofectamine RNAiMAX Transfection Reagent (Thermo Fischer Scientific, Waltham, MA, USA) was applied for siRNA transfection. Proteins were extracted at 48 h after transient transfection for western blot analysis. The sequences of all siRNAs were listed below. si-con sense sequence: UUCUCCGAACGUGUCACGUTT and si-con anti-sense sequence: ACGUGACACGUUCGGAGAATT; si-PRC1-1 sense sequence: GGAGAAUAUUGCAACACUA and si-PRC1-1 anti-sense sequence: UAGUGUUGCAAUAUUCUCC; si-PRC1-2 sense sequence: GAAGCUACUUCAAGAGCAA and si-PRC1-2 anti-sense sequence: UUGCUCUUGAAGUAGCUUC.

### RNA extraction and reverse transcription quantitative PCR (RT-qPCR)

An RNA purification kit (Thermo Fisher Scientific) was applied to extract total RNA. Before reverse transcription, RNA samples were treated with DNase to remove DNA impurities. After that, cDNA was obtained through using the Prime Script RT Reagent Kit (Takara, Tokyo, Japan) to complete reverse transcription of RNA. qPCR was completed on CFX96 Real-Time PCR Detection System (Bio-Rad, Hercules, CA, USA) by applying a SYBR Premix Ex Taq Kit (Yeasen, Shanghai). *β-actin* was used as an internal control gene. Forward primer for *PRC1*, 5′-TAGACCACACCCCAGACACA-3′; reverse primer for *PRC1*, 5′-GTGGCCACAGCTTCTCTTTC-3′. Forward primer for *β-actin*, 5′-CCTGGCACCCAGCACA-3′; reverse primer for *β-actin*, 5′-GCCGATCCACACGGAG-3′.

### Cell viability detection

Briefly, cells were seeded into a 96-well plate at a density of 3 × 10^3^ cells per well and incubated for 48 h. Cells in each well were treated with 10 µl Cell Counting Kit-8 (CCK-8) reagent (Beyotime) for 2 h at 37 °C. Finally, a microplate reader (BD Biosciences, San Jose, CA, USA) was applied to measure the OD450 value.

### Terminal deoxynucleotidyl transferase dUTP nick end labeling (TUNEL) assay

CRC cells (5 × 10^4^/well) were plated on a cover glass in the 24-well plate at ∼70% confluence and incubated for 48 h at 37 °C. After washing thrice in PBS for 10 min, cells were fixed in 2% PFA and stained using the TUNEL kit (Promega, Madison, WI, USA), as per the manufacturer’s protocol. Afterwards, samples were counterstained with DAPI (Solarbio, Beijing, China). TUNEL-positive cells were observed under a laser scanning confocal microscopy (Nikon, Melville, NY, USA) and quantified using the Image J software.

### Immunofluorescence (IF) staining

The *β*-catenin and ICD marker (CRT) were detected by using specific antibodies for IF staining. In brief, cells were seeded onto sterile cover slips and made them adhere overnight. After indicated treatment, cells were fixed with 4% PFA, followed by incubation with the primary antibodies, including anti-*β*-catenin (ab68183; Invitrogen, Carlsbad, CA, USA) and anti-CRT (DF6211; Affinity Biosciences, Changzhou, China) at 4 °C overnight. After that, cells were further incubated with the goat anti-rabbit Fluor 594-conjugated secondary antibody (S0006; Affinity Biosciences) or goat anti-rabbit Fluor 488-conjugated secondary antibody (S0018; Affinity Biosciences) for 1.5 h at 25 °C in the dark. DAPI (Sigma-Aldrich, St. Louis, MO, USA) was used for counterstaining nuclei. The images were captured using a laser scanning confocal microscopy (Nikon) and quantified using the Image J software.

### Measurement of extracellular HMGB1

Enzyme linked immunosorbent assay (ELISA) was applied to assess the release of HMGB1 by CRC cells. Briefly, the supernatants of indicated CRC cells were prepared by centrifugation at 10,000 × g for 3 min at 4 °C. The extracellular level of HMGB1 was measured by using the HMGB1 detection kit (Chondrex, Beijing, China), as per the manufacturer’s protocol.

### Measurement of extracellular ATP level

The release of ATP by indicated CRC cells was measured using ATP Content Assay Kit (Boxbio, Beijing, China) in line with the protocol. Briefly, extracting solution was added into medium and then subjected to centrifugation at 4 °C for 10 min at 10,000 *g*. Supernatant was mixed with chloroform and then centrifuged at 4 °C for 5 min at 10,000 *g*. Supernatant was collected for detection of the absorbance at 340 nm using a microplate reader (BD Biosciences).

### Measurement of cellular ROS production

The cellular ROS production was detected by DCFH-DA (Beyotime) using the ROS assay kit. In brief, cells were treated with 10 µM DCFH-DA in the dark at 37 °C for 30 min. DCFH-DA staining was observed under a fluorescence microscope (Olympus, Tokyo, Japan). A fluorescence microplate reader (BD Biosciences) was applied to analyze the fluorescence intensity at a wavelength pair of 488/525 nm.

### Western blot

Cell lysates were obtained by using RIPA lysis buffer with supplementation of protease inhibitor tablets (Roche, Stockholm, Sweden). Lysates were then subject to SDS-PAGE and transferred onto 10% PVDF membrane (Merck Millipore, Billerica, MA, USA). Proteins on the membranes were then treated with primary antibodies, including anti-PRC1 (ab51248; Abcam, Cambridge, MA, USA), anti-ATF4 (ab270980; Abcam), anti-GRP78 (ab108613; Abcam), anti-CHOP (ab317378; Abcam), anti-PD-L1 (13684; Cell Signaling Technology, Beverly, MA, USA), anti-Wnt1 (200-301-A37; Invitrogen), anti-*β*-catenin (ab68183; Invitrogen), anti-cyclin D1 (DF6386; Affinity Biosciences), anti-Bcl-2 (AF6139; Affinity Biosciences), anti-Bax (AF0120; Affinity Biosciences), and the loading control anti-*β*-actin (AF7018; Affinity Biosciences) for overnight incubation. Afterwards, proteins on the membranes were further incubated with the HRP-conjugation secondary antibodies (S0001, S0002; Affinity Biosciences) for 1 h. The protein bands were detected using the chemiluminescence (ECL™) method and captured by applying a Luminescence Imaging System (Liuyi, Beijing, China).

### Animal study

All animal experimental procedures were reviewed and approved by the Animal Ethics Committee of Cancer Hospital of China Medical University. Before animal experiments, lentiviral particles carrying PRC1-specific short hairpin RNA (sh-PRC1) or its negative control (sh-con) were introduced into SW480 cells to generate stable knockdown cell line. sh-PRC1 and sh-con were obtained from HanBio (Shanghai, China). The sequences of shRNAs were listed below. sh-con sense sequence: TTCTCCGAACGTGTCACGTTT and sh-con anti-sense sequence: ACGTGACACGTTCGGAGAATT); sh-PRC1 sense sequence: GGAGAATATTGCAACACTA and sh-PRC1 anti-sense sequence: ATAGTGTTGCAATATTCTCC. Male BALB/c mice, aged 5 weeks, were commercially provided by Beijing Vital River Laboratory Animal Technology Co., Ltd (Beijing, China). After a week of adaptation, 10 mice were randomly separated into sh-PRC1 and sh-con groups. Each mouse in group one was subcutaneously injected with SW480 cells (1 × 10^6^) with stable transfection of sh-PRC1. The rest mice in the other group were subcutaneously injected with SW480 cells (1 × 10^6^) with stable transfection of sh-con. Tumor growth condition was monitored every 7 days by measuring tumor volume according the formula: volume = 0.5 × length × width^2^. Five weeks later, all mice were sacrificed, and then tumor size and weight were calculated. Tumor tissues were lysed or embedded in paraffin, and then subjected to the subsequent biochemical analysis and histopathological experiments.

### Hematoxylin-eosin (H&E) and immunohistochemistry (IHC) staining

Samples embedded with paraffin were cut into 5-µm sections. Paraffin slides were stained with hematoxylin solution for 5 min and then with eosin for 30 s at 37 °C. Results of H&E staining were observed and imaged under an optical microscope (Olympus). For IHC staining, samples were then subject to antigen retrieval by boiling in a citrate buffer, followed by incubation with anti-Wnt1 (200-301-A37; Invitrogen), anti-CHOP (ab317378; Abcam), anti-PD-L1 (13684; Cell Signaling Technology), anti-Ki67 (BF0132; Affinity Biosciences), anti-CRT (ab195511; Abcam), anti-HMGB1 antibody (6893; Cell Signaling Technology) overnight. Samples were incubated with secondary antibodies (S0001, S0002; Affinity Biosciences) and finally stained with DAB (Nanjing Jiancheng Bioengineering Institute Co., LTD, Nanjing, China). The weak or strong staining were observed and counted under an optical microscope. Quantification was made by using the Image Pro Plus software (Media Cybernetics, Silver Springs, MD, USA). The staining intensity score was defined as 4 levels, which were 0 (negative), 1 (weak), 2 (medium), and 3 (strong). The staining degree was classified according to the percentage of positive staining cells: 0 (0%), 1 (1%~25%), 2 (26%~50%), 3 (51%~75%), and 4 (76%~100%). The product of staining intensity and degree is considered to be the IHC staining score.

### Statistical analysis

The experimental results were displayed as the mean ± standard deviation by using the GraphPad Prism 8.0 (La Jolla, CA, USA). Difference between two groups was compared and analyzed by using the *t* test, while that among three or more groups was compared and analyzed through applying one-way analysis of variance with Tukey’s posthoc test. All statistical analyses were completed by utilizing the SPSS software (IBM, Armonk, NY, USA). *P* < 0.05 is a symbol of statistically significant difference.

## Results

### PRC1 is abnormally overexpressed in CRC and predicts poor patient’s prognosis

**Fig. 1:**
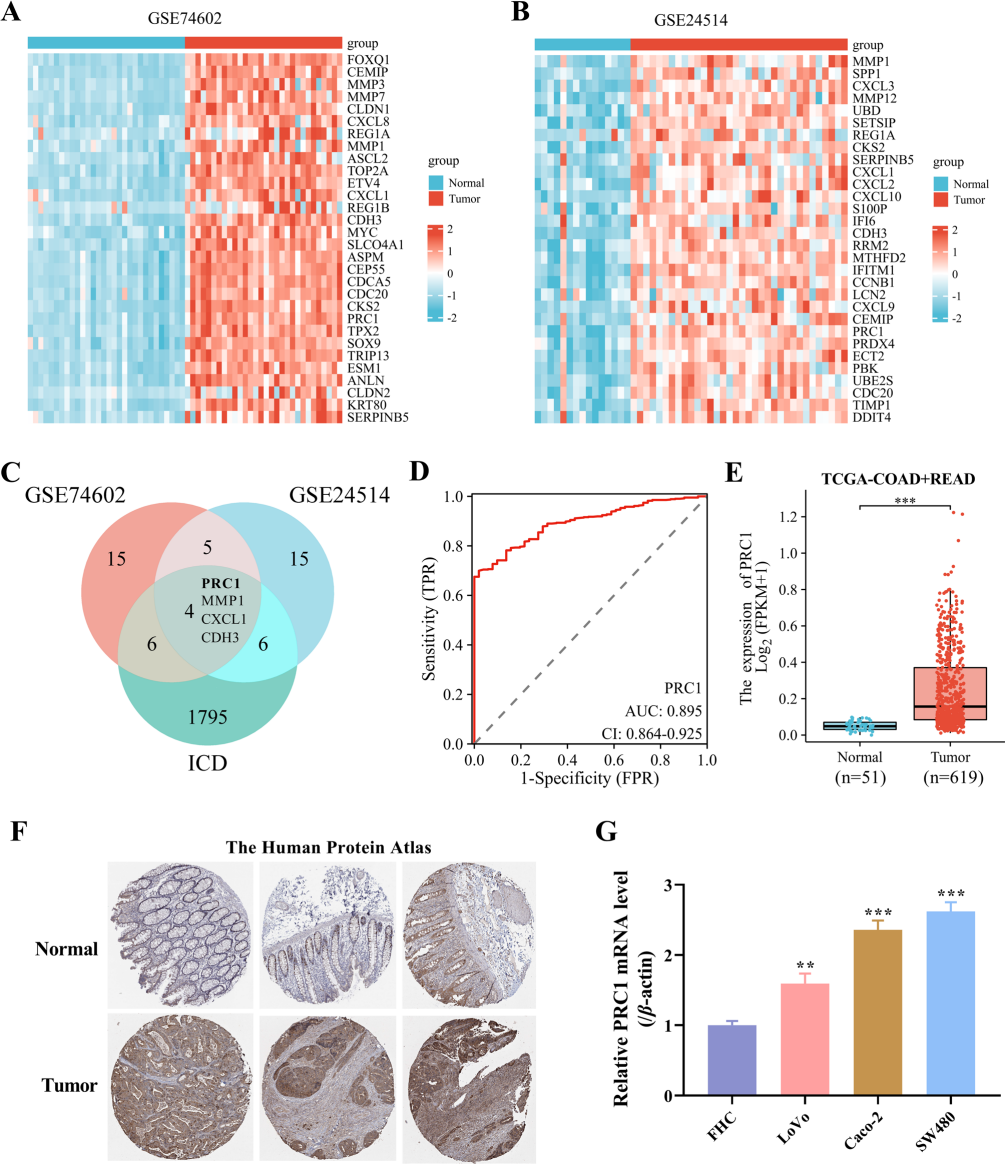
PRC1 is abnormally overexpressed in CRC and predicts poor patients’ prognosiss. (**A**) GSE74602 dataset was analyzed and corresponding heatmap was generate to show top 30 genes significantly upregulated (adjp < 0.05, logFC > = 1) in 30 tumor tissues obtained from CRC patients. (**B**) GSE24514 dataset was analyzed and corresponding heatmap was generate to show top 30 genes significantly upregulated (adjp < 0.05, logFC > = 1) in 34 tumor tissues collected from MSI CRC patients. (**C**) Four candidate mRNAs were obtained by taking the intersection of top 30 upregulated genes in GSE74602 and GSE24514 datasets and 1811 protein coding genes associated with ICD screened out from Genecards. (**D**) The diagnostic validity of PRC1 expression was analyzed by plotting the ROC curve. (**E**) The expression pattern of PRC1 in tumor tissues obtained from both COAD and READ patients and normal colon tissues in TCGA database. (**F**) IHC data obtained from HPA database indicated that positive PRC1 expression was higher in tumor tissues than that in normal tissues collected from COAD and READA patients. (**G**) RT-qPCR data showed that PRC1 presented higher expression in three CRC cells compared with the normal FHC cell. ^**^P < 0.01 and ^***^P < 0.001

At first, two GEO datasets (GSE74602 and GSE24514) were analyzed and corresponding heatmaps were generated. Top 30 genes significantly upregulated (adjp < 0.05, logFC > = 1) in 30 tumor tissues obtained from CRC patients (Fig. 1A) and in 34 tumor tissues collected from MSI CRC patients (Fig. 1B). Agents that induce ICD are used to shape tumor immunogenicity and are recognized as a method for CRC treatment. Thus, a total of 1811 protein coding genes associated with ICD were screened out from Genecards. By taking the intersection of all above three datasets, four candidate mRNAs were obtained (Fig. 1C). Studies have shown the role of PRC1 in CRC [14] and revealed its role as an immune cell marker [15]. Thus, we chose PRC1 as research object for further detection. In addition, the diagnostic validity of PRC1 expression was analyzed by plotting the receiver operating characteristic (ROC) curve. As shown in Fig. 1D, the area under the curve (AUC) of PRC1 was 0.895, indicating that PRC1 was an ideal diagnostic biomarker for CRC. Subsequently, the expression pattern of PRC1 was further identified by analyzing TCGA clinical data. The results indicated that PRC1 presented significant high expression in tumor tissues obtained from both COAD and READ patients (Fig. 1E). Meanwhile, IHC data obtained from HPA database indicated that positive PRC1 expression was higher in tumor tissues than that in normal tissues collected from COAD and READ patients (Fig. 1F). Consistently, PRC1 presented higher expression in CRC cells compared with the normal FHC cell (Fig. 1G), as presented in RT-qPCR data. These results suggest that overexpression of PRC1 is a poor prognostic factor in CRC.

### PRC1 is closely correlated with ICD in CRC

**Fig. 2:**
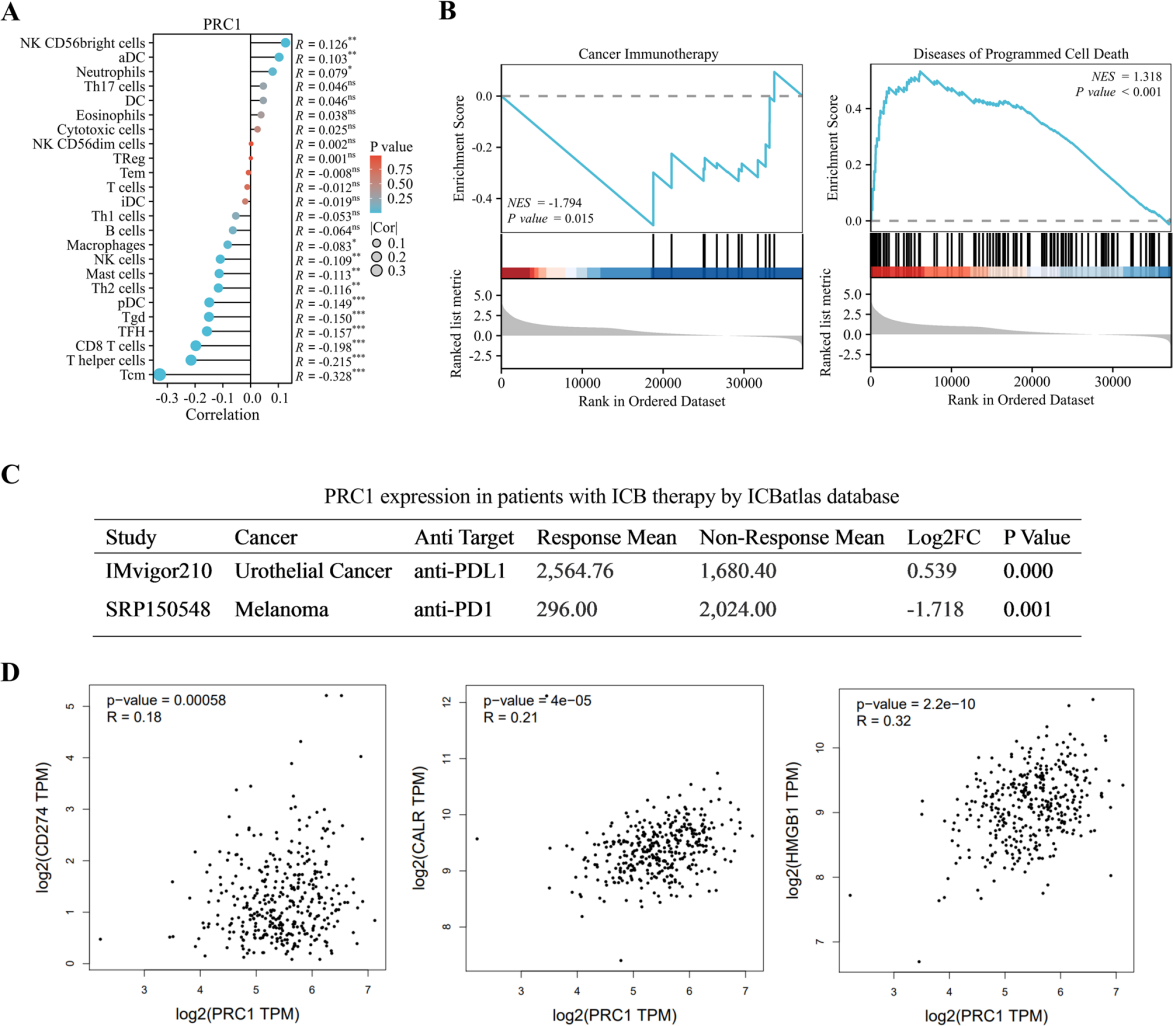
PRC1 is closely correlated with ICD in CRC. (**A**) Correlation analysis of immune cell infiltration revealed that PRC1 had a stable and significant correlation with central memory T cell. (**B**) GSEA single gene differential analysis indicated that PRC1 is related to cancer immunotherapy (left) and PCD (right). (**C**) Data downloaded from ICBatlas showed that PRC1 expression was significantly related to ICB therapy with anti-PDL1 as target. (**D**) GEPIA data indicated that PRC1 had a positive expression correlation with ICD markers, including CD274 (PD-L1), CALR (CRT), and HMGB1 in tumor tissues from COAD and READ patients

The current study further unraveled whether PRC1 had correlation with ICD in CRC. PRC1 had a stable and significant correlation with immune cell infiltration (Fig. 2A). Through GSEA single gene differential analysis, we determined that PRC1 was related to cancer immunotherapy (Fig. 2B, left) and programmed cell death (PCD) (Fig. 2B, right). Since ICD is a kind of PCD, we further supposed that PRC1 might affect ICD. Next, data downloaded from ICBatlas showed that PRC1 expression was significantly related to ICB therapy with anti-PD-L1 as target (Fig. 2C). Moreover, GEPIA data indicated that PRC1 had a positive expression correlation with ICD markers, including CD274 (PD-L1), CALR (CRT), and HMGB1 in tumor tissues from COAD and READ patients (Fig. 2D). Taken together, we confirm the relevance between PRC1 and ICD.

### Knockdown of PRC1 promotes ICD in CRC

**Fig. 3:**
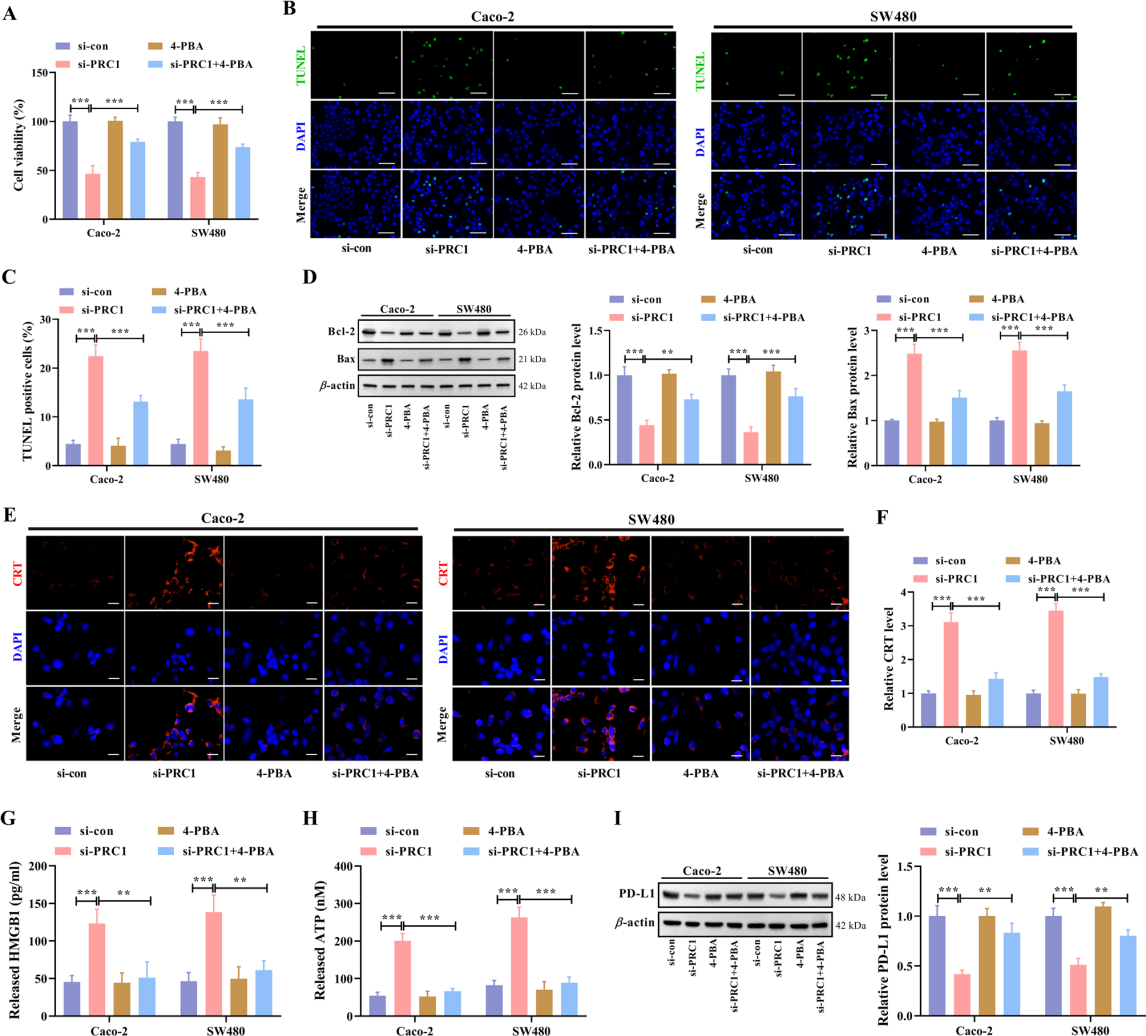
Knockdown of PRC1 promotes ICD in CRC. CRC cells were transfected with si-PRC1 and then treated with 4 mM 4-PBA (ER stress inhibitor) for 48 h. (**A**) Cell viability was determined through CCK-8 detection. (**B**) TUNEL assay was performed to analyze cell apoptosis condition. (**C**) TUNEL-positive cells were counted and analyzed. (**D**) The expression levels of Bcl-2 and Bax proteins were analyzed by western blot. (**E**) CRT expression in two CRC cell lines was detected by IF staining. (**F**) The results of quantitative analysis of fluorescence intensity were presented. (**G**,** H**) Released HMGB1 and ATP levels in culture medium of two CRC cell lines were detected by ELISA and ATP Assay Kit, respectively. (**I**) PD-L1 protein expression in two CRC cell lines was measured by western blot. ^**^P < 0.01 and ^***^P < 0.001

Subsequently, this study unraveled the functions of PRC1 in modulating ICD. Knockdown efficacy of si-PRC1 was evaluated by RT-qPCR assay. PRC1 mRNA levels were significantly reduced in CRC cells transfected with si-PRC1-1 or si-PRC1-2 (Fig. S1). Through CCK-8 detection, cell viability was found to be reduced a lot by PRC1 knockdown, but was strengthened again after treating with the ER stress inhibitor 4-PBA (Fig. 3A). Additionally, PRC1 knockdown led to a significant increase in the apoptosis of two CRC cell lines, while this trend was attenuated after 4-PBA treatment (Fig. 3B, C), as reflected by changes of TUNEL-positive cell percent. A decrease of Bcl-2 (anti-apoptotic protein) level and an increase of Bax (pro-apoptotic protein) level were observed in CRC cells with PRC1 knockdown, and these changes were partially reversed by 4-PBA (Fig. 3D). Furthermore, CRT expression in two CRC cell lines was detected by IF staining after indicated transfection or treatment. The results unveiled that CRT expression was obviously enhanced by PRC1 knockdown, but was then reduced by 4-PBA co-treatment (Fig. 3E, F). Moreover, PRC1 knockdown increased release of HMGB1 in two CRC cell lines, while the increased level was reduced by 4-PBA co-treatment (Fig. 3G). In addition, the extracellular ATP level was found to be increased markedly in PRC1-silenced CRC cells, but was decreased again after 4-PBA co-treatment (Fig. 3H). To further detect the effect of PRC1 on immune checkpoint blockade, PD-L1 protein expression was measured. As shown in western blot data, PD-L1 protein level decreased by PRC1 knockdown was recovered after 4-PBA co-treatment (Fig. 3I). According to all these results, we summarize that PRC1 can suppress ICD in CRC.

### Knockdown of PRC1 promotes ROS-mediated ER stress in CRC cells

**Fig. 4:**
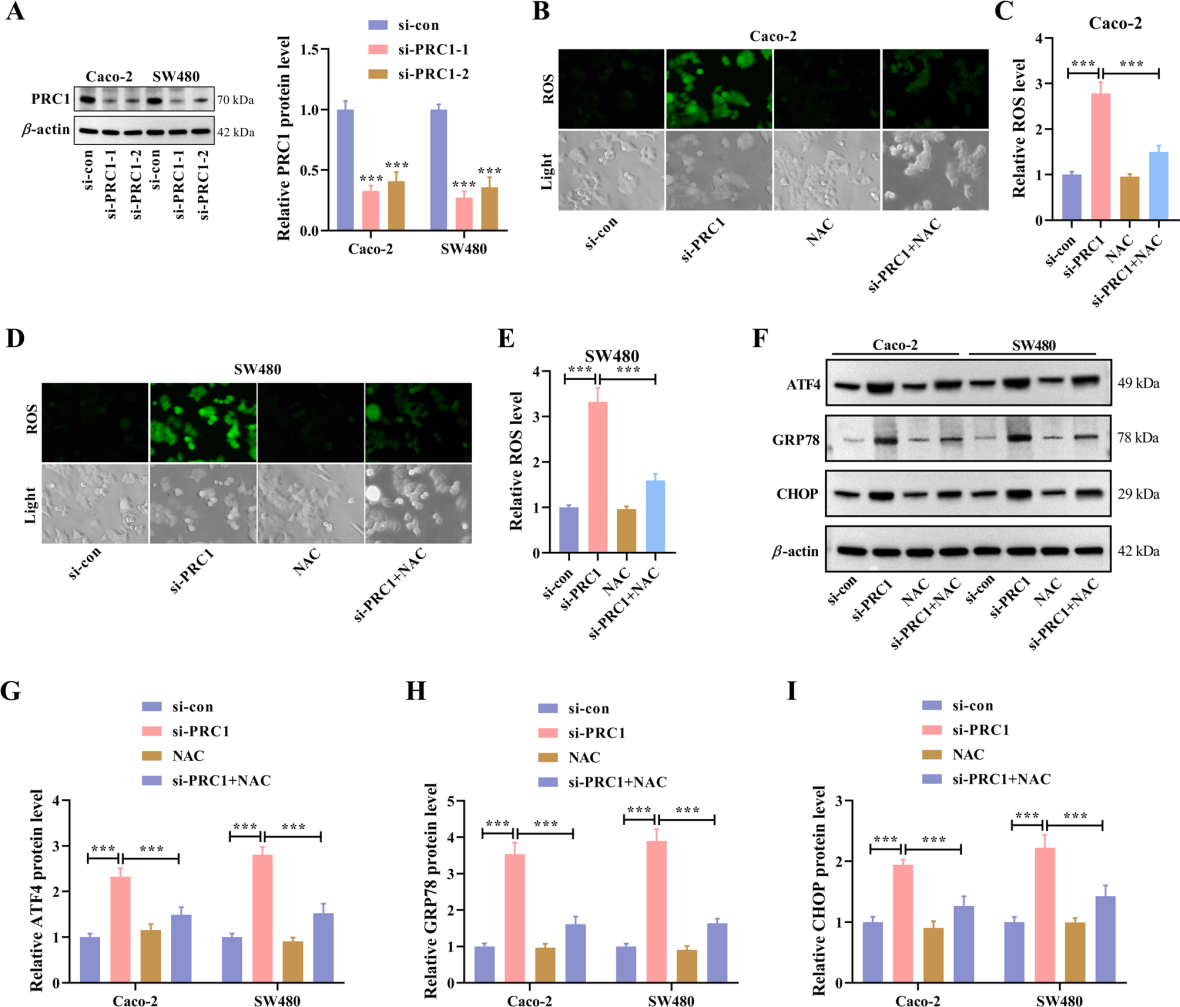
Knockdown of PRC1 promotes ROS-mediated ER stress in CRC cells. (**A**) PRC1 expression was knocked down in two CRC cell lines with higher PRC1 expression (Caco-2 and SW-480). Knockdown efficiency was determined by western blot. (**B-E**) The intracellular ROS production in two CRC cell lines was detected by DCFH-DA staining. Representative fluorescent images were shown on the left. The ROS production was analyzed by a microplate reader and quantitative analysis was conducted. (**F-I**) Western blot and quantification analysis revealed the expression changes in three ER stress-related biomarkers (ATF4, GRP78, and CHOP) in CRC cells with the indicated treatments. ^***^P < 0.001

ER stress-induced apoptosis has similar characteristics with ICD and can trigger a strong immune response against dead-cell antigens [6]. Therefore, the next study focused on the effects of PRC1 on ER stress in CRC. Before that, PRC1 expression was knocked down in two CRC cell lines with higher PRC1 expression (Caco-2 and SW-480). According to western blot results, after transfection with PRC1-specific siRNAs, the protein level of PRC1 was markedly reduced compared with the si-con group (Fig. 4A). NAC has been proven to be capable of inhibiting ER stress through redox regulation [27] and unfolded protein response modulation [28, 29]. PRC1 knockdown increased intracellular ROS production, whereas NAC pretreatment could attenuate PRC1 knockdown-induced ROS generation (Fig. 4B-E, Fig. S2A). ATF4 is a transcription factor and is critical to the ER stress response. GRP78 and CHOP are the downstream effectors of ATF4 and also two ER stress-related biomarkers. Next, western blot was applied to detect the expression changes of abovementioned three ER stress markers. The results revealed that the protein levels of ATF4, GRP78, and CHOP were all elevated significantly by PRC1 knockdown, whereas NAC pretreatment could weaken these tendencies caused by PRC1 knockdown (Fig. 4G-M). Additionally, PRC1 knockdown resulted in elevated protein levels of ATF6 and IRE1 (Fig. S3A-C). The results reveal that PRC1 inhibits ER stress in CRC cells.

### PRC1 activates the Wnt/*β*-catenin signaling pathway in CRC cells

**Fig. 5:**
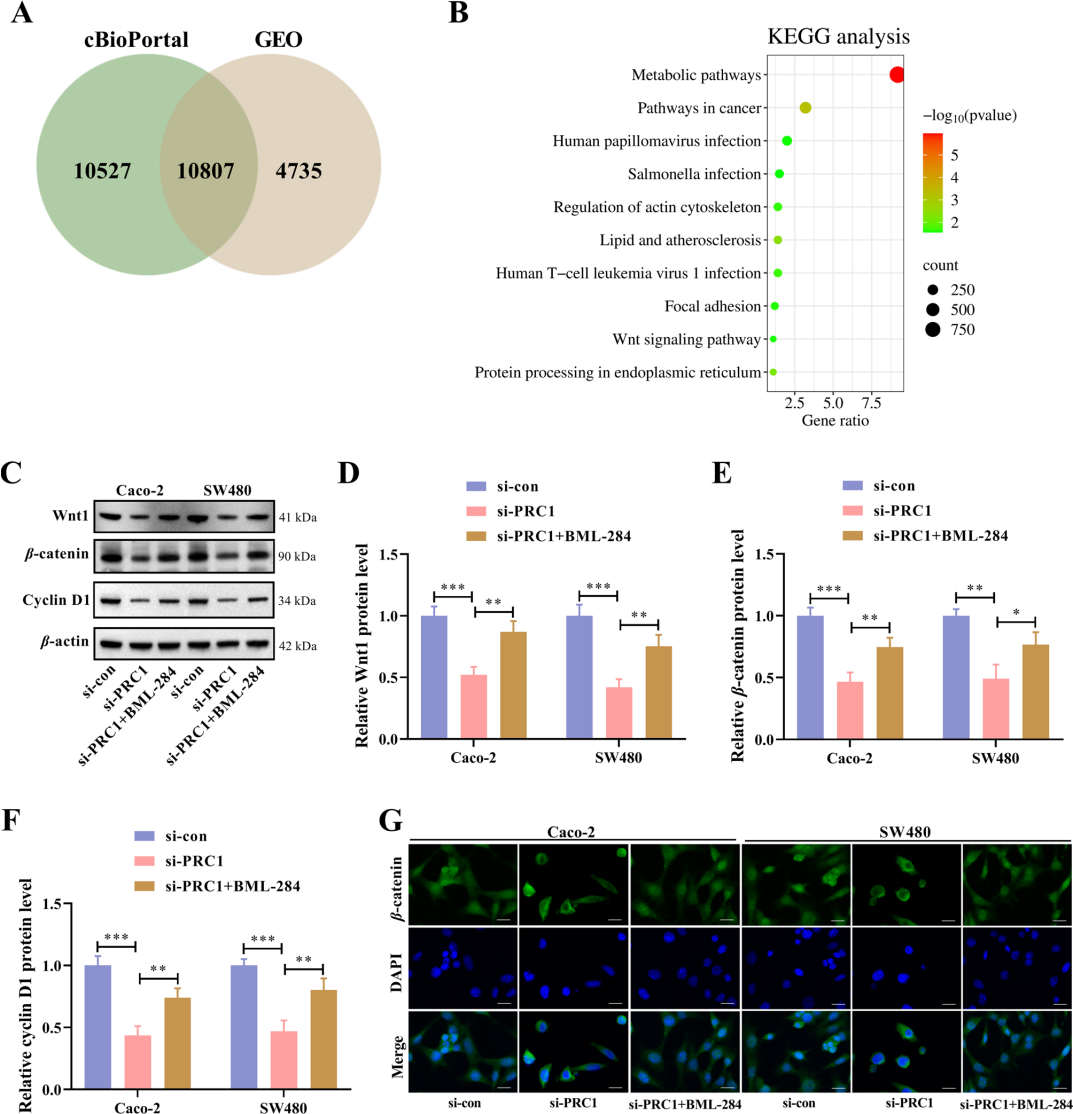
PRC1 activates Wnt/*β*-catenin signaling pathway in CRC cells. (**A**) A total of 10,807 targets were selected out by intersecting 21,334 PRC1-related genes screened from cBioPortal, a total of 15,542 differentially expressed genes found out by analyzing two GEO datasets (GSE74602 and GSE24514). (**B**) A total of 10,807 targets were subject to KEGG analysis to show pathways that PRC1 downstream targets enriched in. (**C-F**) CRC cells were transfected with si-PRC1 and then treated with 0.5 µM BML-284 (Wnt signaling activator) for 48 h. Western blot was conducted to determine the protein levels of Wnt1, *β*-catenin, and cyclin D1. (G) The subcellular localization of *β*-catenin in CRC cells was detected by immunofluorescence assay. ^*^P < 0.05, ^**^P < 0.01, and ^***^P < 0.001

Next, we focused on the downstream mechanism of PRC1 in CRC cells. Through Pan-cancer analysis of whole genomes in cBioPortal, there were 21,334 PRC1-related genes. Moreover, a total of 15,542 differentially expressed genes were found out by analyzing two GEO datasets (GSE74602 and GSE24514). After intersection all above results, 10,807 targets were selected out (Fig. 5A) and subjected to KEGG analysis. Pathways that PRC1 downstream targets enriched in were displayed in Fig. 5B. Among all these pathways, the Wnt/*β*-catenin signaling pathway has been proven to be a potential ICD regulator [26]. In addition, PRC1 has been demonstrated to regulate the Wnt/β-catenin signaling in hepatocellular carcinoma [20]. Thus, western blot was performed to prove whether Wnt/*β*-catenin was the downstream pathway of PRC1. Results demonstrated that protein levels of two core effectors of the Wnt/*β*-catenin signaling pathway (Wnt1 and *β*-catenin) and its downstream gene cyclin D1 were significantly reduced by PRC1 knockdown, while activation of Wnt/*β*-catenin by BML-284 treatment could partially rescued the effects of PRC1 knockdown (Fig. 5C-F). Moreover, knockdown of PRC1 led to a reduction in nuclear *β*-catenin in CRC cells, but these changes were rescued after BML-284 treatment (Fig. 5G). These findings unveil that PRC1 acts as an activator of the Wnt/*β*-catenin signaling pathway in CRC cells.

### Activation of the WNT/*β*-catenin signaling pathway diminishes the effect of PRC1 knockdown on ICD

**Fig. 6:**
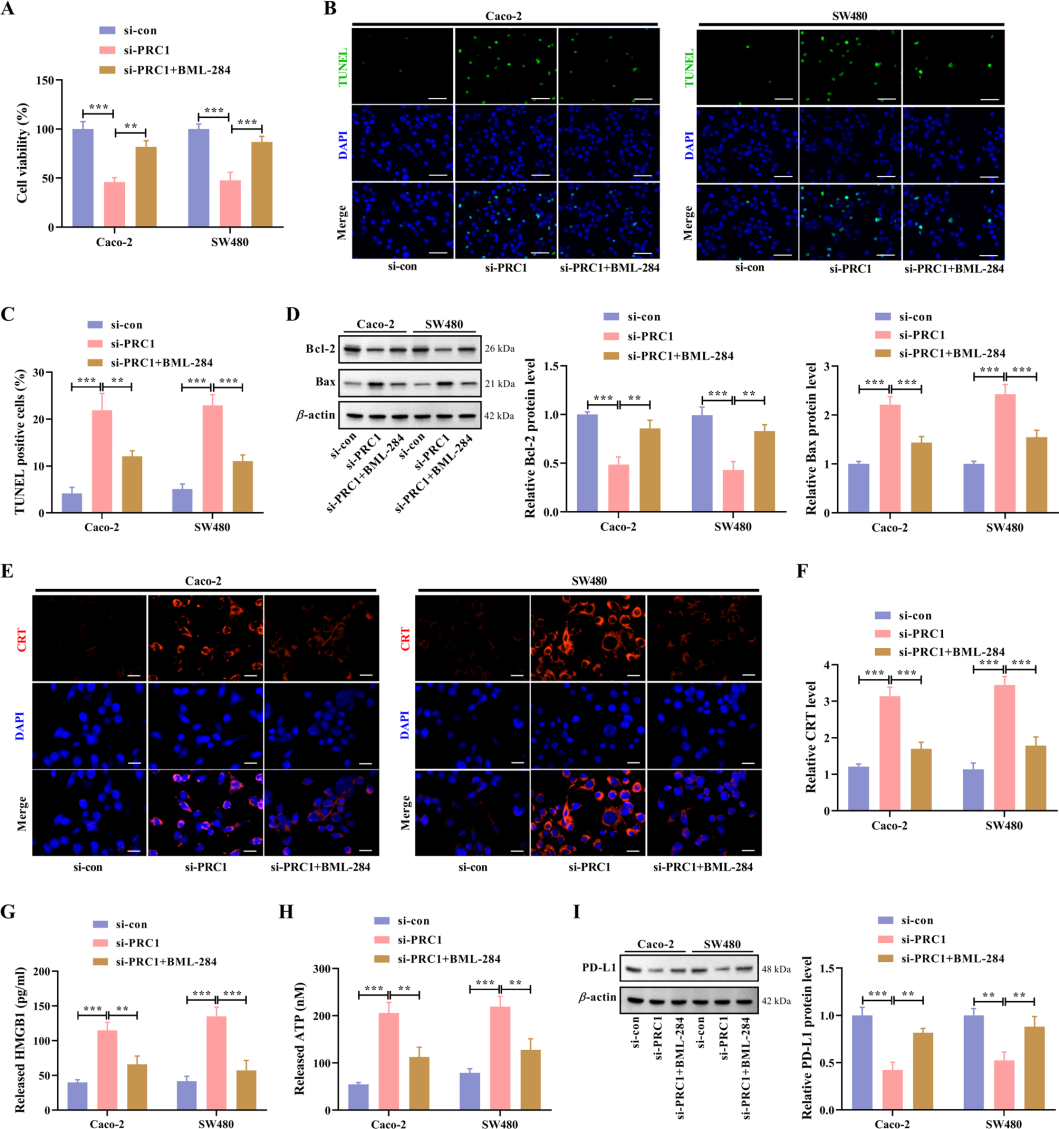
Activation of WNT/*β*-catenin signaling pathway diminishes the effect of PRC1 knockdown on ICD. CRC cells were transfected with si-PRC1 and then treated with 0.5 µM BML-284 for 48 h. (**A**) CCK-8 assay was applied to assess cell viability. (**B**,** C**) Cell apoptosis in two CRC cell lines was evaluated by TUNEL assay. (**D**) The expression levels of Bcl-2 and Bax proteins were analyzed by western blot. (**E**) CRT expression was detected by IF staining and representative images were shown. (**F**) Quantitative analysis was conducted to compare the fluorescence intensity of IF staining. (**G**,** H**) Extracellular HMGB1 and ATP levels were detected by ELISA and ATP Assay Kit, respectively. (**I**) Western blot analysis for the PD-L1 protein level in two CRC cell lines was performed. ^**^P < 0.01 and ^***^P < 0.001

Functionally, BML-284-induced Wnt/*β*-catenin activation recovered cell viability inhibited by PRC1 knockdown (Fig. 6A). In addition, PRC1 knockdown-induced apoptosis in two CRC cell lines could be lessened after BML-284 co-treatment (Fig. 6B-D). The ICD marker CRT was also detected by IF staining. Results showed that CRT expression enhanced by PRC1 knockdown was weakened by BML-284 co-treatment (Fig. 6E, F). Moreover, extracellular HMGB1 and ATP levels increased by PRC1 knockdown were lowered by BML-284 co-treatment (Fig. 6G, H). Western blot data demonstrated that BML-284 treatment restored the PD-L1 protein level decreased by PRC1 knockdown (Fig. 6I). Therefore, we conclude that PRC1 suppresses ICD in CRC via Wnt/*β*-catenin activation.

### Activation of the WNT/*β*-catenin signaling pathway attenuates the effect of PRC1 knockdown on ER stress

**Fig. 7:**
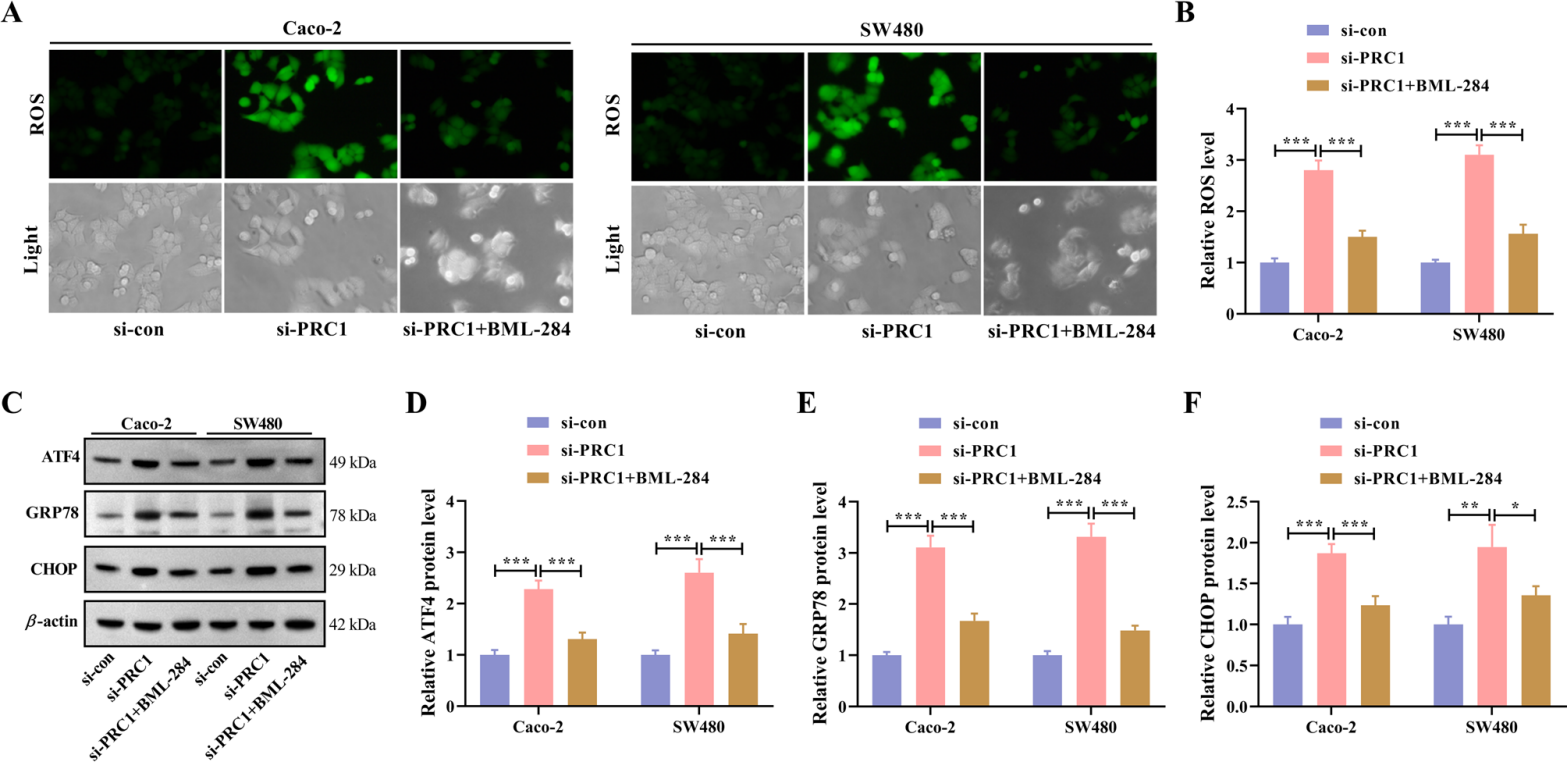
Activation of WNT/*β*-catenin signaling pathway attenuates the effect of PRC1 knockdown on ER stress. CRC cells were transfected with si-PRC1 and then treated with 0.5 µM BML-284 for 48 h. (**A**) Representative fluorescent images of DCFH-DA staining were shown on the left. (**B**) ROS production was analyzed by a microplate reader and quantitative analysis was conducted. (**C**) Western blot analysis for three ER stress-related biomarkers (ATF4, GRP78, and CHOP) was conducted. (**D-F**) Quantitative results of protein bands were shown. ^*^P < 0.05, ^**^P < 0.01, and ^***^P < 0.001

In subsequence, we continued to analyze whether PRC1 can exert functions via the Wnt/*β*-catenin signaling pathway. Results showed that the increased ROS production caused by PRC1 knockdown was reduced again after treating with BML-284 to activate Wnt/*β*-catenin (Fig. 7A, B, Fig. S2B). Moreover, BML-284 treatment-induced Wnt/*β*-catenin activation abolished the promoting effects of PRC1 silencing on the protein levels of ATF4, GRP78, and CHOP in two CRC cell lines (Fig. 7C-F). In summary, PRC1 can inhibit ER stress in CRC cells via activation of the Wnt/*β*-catenin signaling pathway.

### PRC1 Silencing restrains tumor growth in CRC animal models

**Fig. 8:**
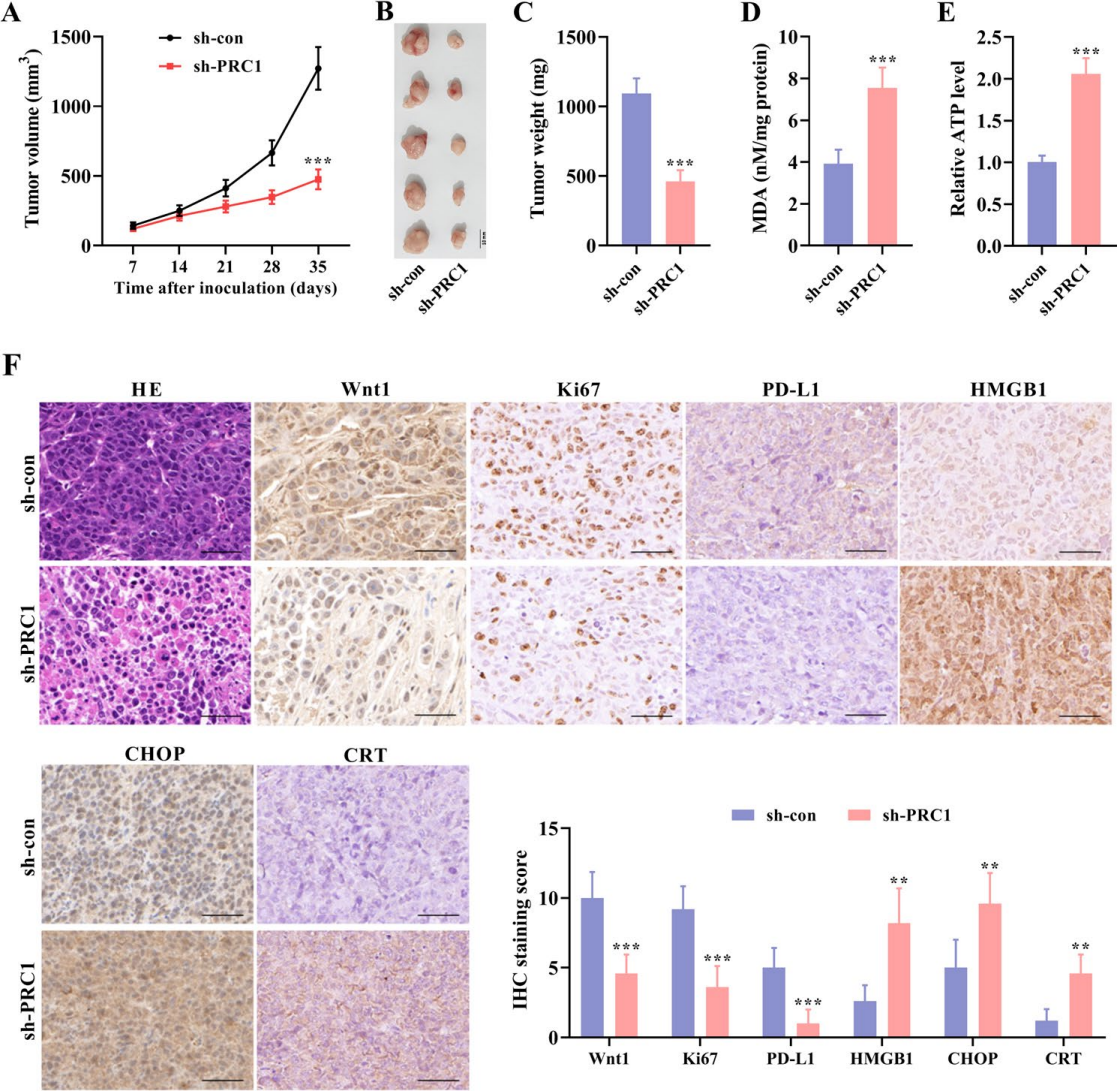
PRC1 silencing enhances anti-tumor efficacy in CRC animal models. Animal models were established to validate the effects of PRC1 on in vivo tumor growth. Five weeks after subcutaneous tumor transplantation, tumors in each group were weighed and measured. (**A**) Tumor growth curve was generated to show tumor growth condition in two groups of mice (sh-con and sh-PRC1). (**B**,** C**) Tumor size and tumor weight in PRC1 silencing group and control group were calculated and shown. (**D**,** E**) The MDA and ATP levels in transplanted tumors were evaluated using their corresponding commercial kits. (**F**) H&E staining showed the histopathological changes in tumor tissues. IHC was conducted to assess the protein expression of Wnt1, proliferation marker (Ki-67), PD-L1, ER stress-related biomarker (CHOP), and two ICD markers (CRT and HMGB1) in tumor tissues. ^**^P < 0.01 and ^***^P < 0.001

Animal models were established to validate the effects of PRC1 on in vivo tumor growth. Five weeks after subcutaneous tumor transplantation, tumors in each group were weighed and measured. It was uncovered that PRC1 silencing led to significant suppression on tumor growth (Fig. 8A), which was also reflected by the smaller tumor size and lighter tumor weight in the PRC1 silencing group (Fig. 8B, C). Additionally, the MDA content in the sh-PRC1 group was more than that in the sh-con group (Fig. 8D). Similarly, the ATP level was higher in the sh-PRC1 group compared with the sh-con group (Fig. 8E). As shown by H&E staining, compared with the sh-con group, the sh-PRC1 group exhibited nuclear shrinkage and structural disorder, as well as more apoptotic and necrotic cells. IHC data revealed that the positive expression levels of Wnt1, proliferation marker (Ki-67), and PD-L1 were significantly lower in the sh-PRC1 group than those in the sh-con group. Elevated positive expression levels of ER stress-related biomarker (CHOP) and two ICD markers (HMGB1 and CRT) were observed in the sh-PRC1 group than those in the sh-con group (Fig. 8F). Collectively, PRC1 can promote tumor growth in CRC.

## Discussion

According to previous findings, PRC1 is regarded as an oncoprotein in diverse human cancers such as oral cancer [30], breast cancer [31], and hepatocellular carcinoma [32]. Consistent with previous reports, we reconfirm that PRC1 is an oncogene, evidenced by reduced cell activity and increased apoptosis in PRC1-silenced CRC cells. Based on bioinformatics analyses, the ectopically expressed PRC1 was identified as a potential diagnostic biomarker for CRC and was closely correlated with ICD in CRC. Previous studies have revealed the relevance of PRC1 with immunotherapy [16, 17] and immune suppression [18]. Increasing evidences have shown that therapy-induced ICD can efficiently enhance tumor immune surveillance via acting as ‘in situ vaccines’ [33–35]. Combination of agents or drugs inducing ICD and ICB therapy has been proven to be more efficient in MSI CRC [36]. PD-L1 (CD274) usually sends a signal of “don’t find me” to the adaptive immune system [37]. Upregulation of PD-L1 in tumor cells weakens the activity of immune cells, thus leading to immune escape [38]. In this study, we determined that PRC1 expression was significantly related to ICB therapy with anti-PD-L1 as target in accordance with data downloaded from ICBatlas. We then found from GEPIA database that PRC1 had a positive expression correlation with ICD markers (CRT and HMGB1) and the immune checkpoint gene CD274 in tumor tissues from COAD and READ patients. Further experimental results showed that PRC1-silenced CRC cells exhibited DAMPs, evidenced by the surge of CRT protein in plasma membrane and increased extracellular release of ATP and HMGB1. Moreover, we confirmed that PRC1 knockdown blocked PD-L1 expression in CRC cells. Our study is the first to experimentally identify the ICD-regulating function of PRC1 in CRC cells.

The increasing translocation of CRT from ER to the cell surface is a cardinal hallmark of ICD [39]. ER stress can induce the ROS generation [40], the latter plays crucial role in ER stress-induced cell death [41]. ROS-mediated ER stress has been demonstrated to one of the key inducements of ICD in hepatocellular carcinoma [42]. In our study, we demonstrated that ICD was triggered in PRC1-silenced CRC cells, while this surge was reversed by the ER stress inhibitor 4-PBA. Additionally, inhibition of ER stress weakened the downregulation of PD-L1 expression induced by PRC1 knockdown, implying that PRC1 regulated ER stress-mediated ICD via PD-L1 in CRC cells. Further experimental research uncovered that knockdown of PRC1 could promote ER stress in CRC cells, while NAC retarded this tendency, suggesting that the regulation of ER stress by PRC1 was at least partially attributed to its promoting effect on ROS generation.

Wnt/*β*-catenin is a classical oncogenic signaling pathway which has been reported in diverse human cancer types [43, 44], including CRC [45]. Evidence has proven the role of Wnt/*β*-catenin in upregulating the expression of PD-L1 protein in cancer cells and thus assisting cancer cells to escape immune surveillance [46]. Targeting Wnt/*β*-catenin could promote ICD in melanoma [26]. However, whether PRC1 could regulate Wnt/*β*-catenin and interfere with ICD in CRC has not been reported. In our investigation, PRC1 knockdown exerted suppressive role in the protein levels of Wnt/*β*-catenin core effectors in CRC cells, indicating that PRC1 acted as an activator of Wnt/*β*-catenin signaling pathway. In subsequence, our study revealed that activation of Wnt/*β*-catenin by BML-284 could effectively reverse the effect of PRC1 knockdown on ICD, ER stress, as well as PD-L1 expression in CRC cells. These findings imply that inhibition of PRC1 might avoid immune escape in CRC through Wnt/*β*-catenin-dependent PD-L1 expression suppression.

In conclusion, our data corroborated that PRC1 silencing could evoke ER stress-dependent ICD in CRC cells via inactivating the Wnt/*β*-catenin signaling pathway. We elucidate a novel molecular pathway whereby PRC1 exerts carcinogenic role in tumor immune microenvironment through ICD in CRC. Our findings highlight the potential of PRC1 as a therapeutic target for the ICB therapy of CRC.

## Supplementary Information

Below is the link to the electronic supplementary material.

Supplementary Material 1

## Data Availability

No datasets were generated or analysed during the current study.

## Abbreviations

APCs Antigen: presenting cells
AUC: Area under the curve
CCK-8: Cell Counting Kit-8
CRC: Colorectal cancer
CRT: Chaperone calreticulin
DAMPs: Damage-associated molecular patterns
ELISA: Enzyme linked immunosorbent assay
ER: Endoplasmic reticulum
FBS: Fetal bovine serum
GEO: Gene Expression Omnibus
GSEA: Gene set enrichment analysis
GTEx: Genotype-Tissue Expression
H&E: Hematoxylin-eosin
HMGB1: High-mobility group box 1
ICB: Immune checkpoint blockade
ICD: Immunogenic cell death
IF: Immunofluorescence
IHC: Immunohistochemistry
MSI: Microsatellite instability
MAPs: Microtubule-associated proteins
NAC: N-acetylcysteine
PCD: Programmed cell death
PRC1: Protein regulator of cytokinesis 1
ROC: Receiver operating characteristic
RT-qPCR: RNA extraction and reverse transcription quantitative PCR
siRNA: Small interfering RNA
sh-con: Short hairpin RNA control
sh-PRC1: Lentiviral particles carrying PRC1-specific short hairpin RNA
TCGA: The Cancer Genome Atlas
TUNEL: Terminal deoxynucleotidyl transferase dUTP nick end labeling

## References

[1] Murphy CC, Zaki TA. Changing epidemiology of colorectal cancer - birth cohort effects and emerging risk factors. 2024;21(1):25–34. 10.1038/s41575-023-00841-9 37723270

[2] Ganesh K, Stadler ZK, Cercek A, Mendelsohn RB, Shia J, Segal NH, Diaz LA. Jr. Immunotherapy in colorectal cancer: rationale, challenges and potential. Nat Rev Gastroenterol Hepatol. 2019;16(6):361–75. 30886395 10.1038/s41575-019-0126-x PMC7295073

[3] Pitt JM, Vétizou M, Daillère R, Roberti MP, Yamazaki T, Routy B, Lepage P, Boneca IG, Chamaillard M, Kroemer G, et al. Resistance mechanisms to Immune-Checkpoint Blockade in cancer: Tumor-Intrinsic and -Extrinsic factors. Immunity. 2016;44(6):1255–69. 27332730 10.1016/j.immuni.2016.06.001

[4] Tumeh PC, Harview CL, Yearley JH, Shintaku IP, Taylor EJ, Robert L, Chmielowski B, Spasic M, Henry G, Ciobanu V, et al. PD-1 Blockade induces responses by inhibiting adaptive immune resistance. Nature. 2014;515(7528):568–71. 25428505 10.1038/nature13954 PMC4246418

[5] Galon J, Bruni D. Approaches to treat immune hot, altered and cold tumours with combination immunotherapies. Nat Rev Drug Discov. 2019;18(3):197–218. 30610226 10.1038/s41573-018-0007-y

[6] Pozzi C, Cuomo A, Spadoni I, Magni E, Silvola A, Conte A, Sigismund S, Ravenda PS, Bonaldi T, Zampino MG, et al. The EGFR-specific antibody cetuximab combined with chemotherapy triggers Immunogenic cell death. Nat Med. 2016;22(6):624–31. 27135741 10.1038/nm.4078

[7] Hossain DMS, Javaid S, Cai M, Zhang C, Sawant A, Hinton M, Sathe M, Grein J, Blumenschein W, Pinheiro EM, et al. Dinaciclib induces Immunogenic cell death and enhances anti-PD1-mediated tumor suppression. J Clin Invest. 2018;128(2):644–54. 29337311 10.1172/JCI94586 PMC5785250

[8] Kroemer G, Galluzzi L, Kepp O, Zitvogel L. Immunogenic cell death in cancer therapy. Annu Rev Immunol. 2013;31:51–72. 23157435 10.1146/annurev-immunol-032712-100008

[9] Rufo N, Garg AD, Agostinis P. The unfolded protein response in Immunogenic cell death and cancer immunotherapy. Trends Cancer. 2017;3(9):643–58. 28867168 10.1016/j.trecan.2017.07.002

[10] Krysko DV, Garg AD, Kaczmarek A, Krysko O, Agostinis P, Vandenabeele P. Immunogenic cell death and damps in cancer therapy. Nat Rev Cancer. 2012;12(12):860–75. 23151605 10.1038/nrc3380

[11] Oresta B, Pozzi C, Braga D, Hurle R, Lazzeri M, Colombo P, Frego N, Erreni M, Faccani C, Elefante G, et al. Mitochondrial metabolic reprogramming controls the induction of Immunogenic cell death and efficacy of chemotherapy in bladder cancer. Sci Transl Med. 2021;13:575. 10.1126/scitranslmed.aba6110 33408185

[12] Pfirschke C, Engblom C, Rickelt S, Cortez-Retamozo V, Garris C, Pucci F, Yamazaki T, Poirier-Colame V, Newton A, Redouane Y, et al. Immunogenic chemotherapy sensitizes tumors to checkpoint Blockade therapy. Immunity. 2016;44(2):343–54. 26872698 10.1016/j.immuni.2015.11.024 PMC4758865

[13] Shrestha S, Wilmeth LJ, Eyer J, Shuster CB. PRC1 controls spindle polarization and recruitment of cytokinetic factors during monopolar cytokinesis. Mol Biol Cell. 2012;23(7):1196–207. 22323288 10.1091/mbc.E11-12-1008 PMC3315816

[14] Xu T, Wang X, Jia X, Gao W, Li J, Gao F, Zhan P, Ji W. Overexpression of protein regulator of cytokinesis 1 facilitates tumor growth and indicates unfavorable prognosis of patients with colon cancer. Cancer Cell Int. 2020;20(1):528. 33292244 10.1186/s12935-020-01618-9 PMC7603724

[15] Pan J, Huang Z, Zhang Y, Xu Y. ADAM12 as a clinical prognostic indicator associated with tumor immune infiltration in lung adenocarcinoma. DNA Cell Biol. 2022;41(4):410–23. 35377217 10.1089/dna.2021.0764

[16] Zhang F, Cai J, Hu K, Liu W, Lu S, Tang B, Li M, Wu W, Ren Z, Yin X. An Immune-Related gene signature predicting prognosis and immunotherapy response in hepatocellular carcinoma. Comb Chem High Throughput Screen. 2022;25(13):2203–16. 35249477 10.2174/1386207325666220304115006

[17] Zhu P, Cui N, Song ZY, Yong WX, Luo XX, Wang GC, Wang X, Wu YN, Xu Q, Zhang LM, et al. PRC1 plays an important role in lung adenocarcinoma and is potentially targeted by fostamatinib. Eur Rev Med Pharmacol Sci. 2022;26(23):8924–34. 36524512 10.26355/eurrev_202212_30567

[18] Zhang C, Xu H, Sui X, Wu T, Chen B, Wang S, Wang X. Protein regulator of cytokinesis 1 (PRC1) upregulation promotes immune suppression in liver hepatocellular carcinoma. J Immunol Res. 2022;2022:7073472. 35983074 10.1155/2022/7073472 PMC9381293

[19] Luo HW, Chen QB, Wan YP, Chen GX, Zhuo YJ, Cai ZD, Luo Z, Han ZD, Liang YX, Zhong WD. Protein regulator of cytokinesis 1 overexpression predicts biochemical recurrence in men with prostate cancer. Biomed Pharmacother. 2016;78:116–20. 26898432 10.1016/j.biopha.2016.01.004

[20] Chen J, Rajasekaran M, Xia H, Zhang X, Kong SN, Sekar K, Seshachalam VP, Deivasigamani A, Goh BK, Ooi LL, et al. The microtubule-associated protein PRC1 promotes early recurrence of hepatocellular carcinoma in association with the Wnt/β-catenin signalling pathway. Gut. 2016;65(9):1522–34. 26941395 10.1136/gutjnl-2015-310625 PMC5036256

[21] Galluzzi L, Buqué A, Kepp O, Zitvogel L, Kroemer G. Immunological effects of conventional chemotherapy and targeted anticancer agents. Cancer Cell. 2015;28(6):690–714. 26678337 10.1016/j.ccell.2015.10.012

[22] Liu J, Xiao Q, Xiao J, Niu C, Li Y, Zhang X, Zhou Z, Shu G, Yin G. Wnt/β-catenin signalling: function, biological mechanisms, and therapeutic opportunities. Signal Transduct Target Ther. 2022;7(1):3. 34980884 10.1038/s41392-021-00762-6 PMC8724284

[23] Zhao H, Ming T, Tang S, Ren S, Yang H, Liu M, Tao Q, Xu H. Wnt signaling in colorectal cancer: pathogenic role and therapeutic target. Mol Cancer. 2022;21(1):144. 35836256 10.1186/s12943-022-01616-7 PMC9281132

[24] Pai SG, Carneiro BA, Mota JM, Costa R, Leite CA, Barroso-Sousa R, Kaplan JB, Chae YK, Giles FJ. Wnt/beta-catenin pathway: modulating anticancer immune response. J Hematol Oncol. 2017;10(1):101. 28476164 10.1186/s13045-017-0471-6 PMC5420131

[25] Zhou Y, Xu J, Luo H, Meng X, Chen M, Zhu D. Wnt signaling pathway in cancer immunotherapy. Cancer Lett. 2022;525:84–96. 34740608 10.1016/j.canlet.2021.10.034

[26] Antony F, Kang X, Pundkar C, Wang C, Mishra A, Chen P, Babu RJ, Suryawanshi A. Targeting β-catenin using XAV939 nanoparticle promotes Immunogenic cell death and suppresses conjunctival melanoma progression. Int J Pharm. 2023;640:123043. 37172631 10.1016/j.ijpharm.2023.123043 PMC10399699

[27] Jiang J, Li D, Li F, Li H, Zhang X, Feng L. Catechin promotes Endoplasmic reticulum stress-mediated gastric cancer cell apoptosis via NOX4-induced reactive oxygen species. Mol Cell Biochem. 2025;480(5):3201–15. 39565530 10.1007/s11010-024-05138-2

[28] Tsai CC, Chen YJ, Yu HR, Huang LT, Tain YL, Lin IC, Sheen JM, Wang PW, Tiao MM. Long term N-acetylcysteine administration rescues liver steatosis via Endoplasmic reticulum stress with unfolded protein response in mice. Lipids Health Dis. 2020;19(1):105. 32450865 10.1186/s12944-020-01274-y PMC7249367

[29] Saha D, Paul S, Gaharwar U, Priya A, Neog A, Singh A, Bk B. Cdk5-Mediated brain unfolded protein response upregulation associated with cognitive impairments in type 2 diabetes and ameliorative action of NAC. 2023;14(15):2761–74. 10.1021/acschemneuro.3c00341 37468304

[30] Wu F, Shi X, Zhang R, Tian Y, Wang X, Wei C, Li D, Li X, Kong X, Liu Y, et al. Regulation of proliferation and cell cycle by protein regulator of cytokinesis 1 in oral squamous cell carcinoma. Cell Death Dis. 2018;9(5):564. 29752448 10.1038/s41419-018-0618-6 PMC5948203

[31] Shimo A, Nishidate T, Ohta T, Fukuda M, Nakamura Y, Katagiri T. Elevated expression of protein regulator of cytokinesis 1, involved in the growth of breast cancer cells. Cancer Sci. 2007;98(2):174–81. 17233835 10.1111/j.1349-7006.2006.00381.x PMC11159940

[32] Liu X, Li Y, Meng L, Liu XY, Peng A, Chen Y, Liu C, Chen H, Sun S, Miao X, et al. Reducing protein regulator of cytokinesis 1 as a prospective therapy for hepatocellular carcinoma. Cell Death Dis. 2018;9(5):534. 29748662 10.1038/s41419-018-0555-4 PMC5945625

[33] Jiang M, Zeng J, Zhao L, Zhang M, Ma J, Guan X, Zhang W. Chemotherapeutic drug-induced Immunogenic cell death for nanomedicine-based cancer chemo-immunotherapy. Nanoscale. 2021;13(41):17218–35. 34643196 10.1039/d1nr05512g

[34] Sun T, Li Y, Yang Y, Liu B, Cao Y, Yang W. Enhanced radiation-induced Immunogenic cell death activates chimeric antigen receptor T cells by targeting CD39 against glioblastoma. Cell Death Dis. 2022;13(10):875. 36245000 10.1038/s41419-022-05319-1 PMC9573869

[35] Shalhout SZ, Miller DM, Emerick KS, Kaufman HL. Therapy with oncolytic viruses: progress and challenges. Nat Rev Clin Oncol. 2023;20(3):160–77. 36631681 10.1038/s41571-022-00719-w

[36] Fang Y, Sun H, Xiao X, Tang M, Tian Z, Wei H, Sun R, Zheng X. Low-dose Immunogenic chemotherapeutics promotes immune checkpoint Blockade in microsatellite stability colon cancer. Front Immunol. 2022;13:1040256. 36389751 10.3389/fimmu.2022.1040256 PMC9647086

[37] Iwai Y, Ishida M, Tanaka Y, Okazaki T, Honjo T, Minato N. Involvement of PD-L1 on tumor cells in the escape from host immune system and tumor immunotherapy by PD-L1 Blockade. Proc Natl Acad Sci U S A. 2002;99(19):12293–7. 12218188 10.1073/pnas.192461099 PMC129438

[38] Miao Z, Li J, Wang Y, Shi M, Gu X, Zhang X, Wei F, Tang X, Zheng L, Xing Y. Hsa_circ_0136666 stimulates gastric cancer progression and tumor immune escape by regulating the miR-375/PRKDC axis and PD-L1 phosphorylation. Mol Cancer. 2023;22(1):205. 38093288 10.1186/s12943-023-01883-y PMC10718020

[39] Galluzzi L, Vitale I, Warren S, Adjemian S, Agostinis P, Martinez AB, Chan TA, Coukos G, Demaria S, Deutsch E et al. Consensus guidelines for the definition, detection and interpretation of Immunogenic cell death. J Immunother Cancer 2020;8(1). 10.1136/jitc-2019-000337 PMC7064135 32209603

[40] He J, Ma M, Li D, Wang K, Wang Q, Li Q, He H, Zhou Y, Li Q, Hou X, et al. Sulfiredoxin-1 attenuates injury and inflammation in acute pancreatitis through the ROS/ER stress/cathepsin B axis. Cell Death Dis. 2021;12(7):626. 34140464 10.1038/s41419-021-03923-1 PMC8211864

[41] Dong L, Xu M, Li Y, Xu W, Wu C, Zheng H, Xiao Z, Sun G, Ding L, Li X, et al. SMURF1 attenuates Endoplasmic reticulum stress by promoting the degradation of KEAP1 to activate NRF2 antioxidant pathway. Cell Death Dis. 2023;14(6):361. 37316499 10.1038/s41419-023-05873-2 PMC10267134

[42] Xu Z, Xu J, Sun S, Lin W, Li Y, Lu Q, Li F, Yang Z, Lu Y, Liu W. Mecheliolide elicits ROS-mediated ERS driven Immunogenic cell death in hepatocellular carcinoma. Redox Biol. 2022;54:102351. 35671636 10.1016/j.redox.2022.102351 PMC9168183

[43] Tian S, Peng P, Li J, Deng H, Zhan N, Zeng Z, Dong W. SERPINH1 regulates EMT and gastric cancer metastasis via the Wnt/β-catenin signaling pathway. Aging. 2020;12(4):3574–93. 32091407 10.18632/aging.102831 PMC7066881

[44] Huang G, Liang M, Liu H, Huang J, Li P, Wang C, Zhang Y, Lin Y, Jiang X. CircRNA hsa_circrna_104348 promotes hepatocellular carcinoma progression through modulating miR-187-3p/RTKN2 axis and activating Wnt/β-catenin pathway. Cell Death Dis. 2020;11(12):1065. 33311442 10.1038/s41419-020-03276-1 PMC7734058

[45] Zhang M, Weng W, Zhang Q, Wu Y, Ni S, Tan C, Xu M, Sun H, Liu C, Wei P, et al. The LncRNA NEAT1 activates Wnt/β-catenin signaling and promotes colorectal cancer progression via interacting with DDX5. J Hematol Oncol. 2018;11(1):113. 30185232 10.1186/s13045-018-0656-7 PMC6125951

[46] Tang Y, Nan N, Gui C, Zhou X, Jiang W, Zhou X. Blockage of PD-L1 by FERMT3-mediated Wnt/β-catenin signalling regulates chemoresistance and immune evasion of colorectal cancer cells. Clin Exp Pharmacol Physiol. 2022;49(9):988–97. 35672907 10.1111/1440-1681.13685

