# Supplementary material for: Inhibition of PRC1 elicits immunogenic cell death by triggering ROS-dependent ER stress in colorectal cancer via the Wnt/β-catenin signaling pathway
Source: Biol Direct. 2025 Aug 22;20:94. doi: 10.1186/s13062-025-00685-0 (PMC12372356; doi:10.1186/s13062-025-00685-0)
Supplement: Supplementary file 1 — Supplementary Material 1 [file 13062_2025_685_MOESM1_ESM.docx]

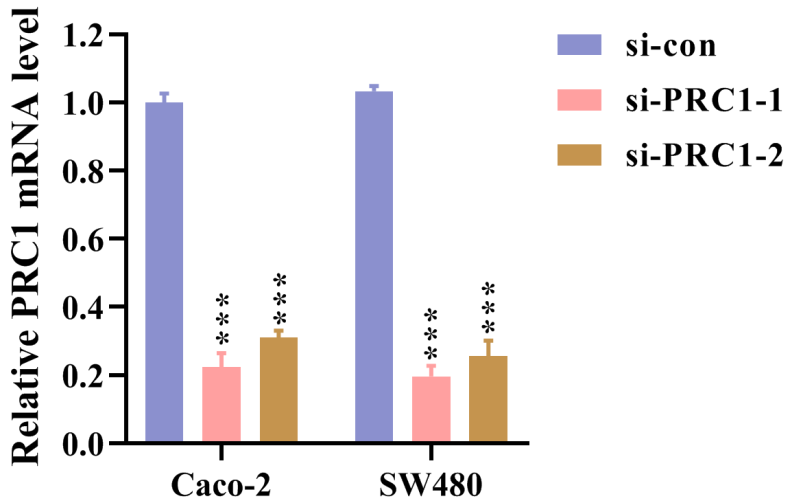


Fig. S1. Knockdown efficacy of si-PRC1 is evaluated by RT-qPCR assay. PRC1 mRNA levels were significantly reduced in CRC cells transfected with si-PRC1-1 or si-PRC1-2. ^***^*P* < 0.001.


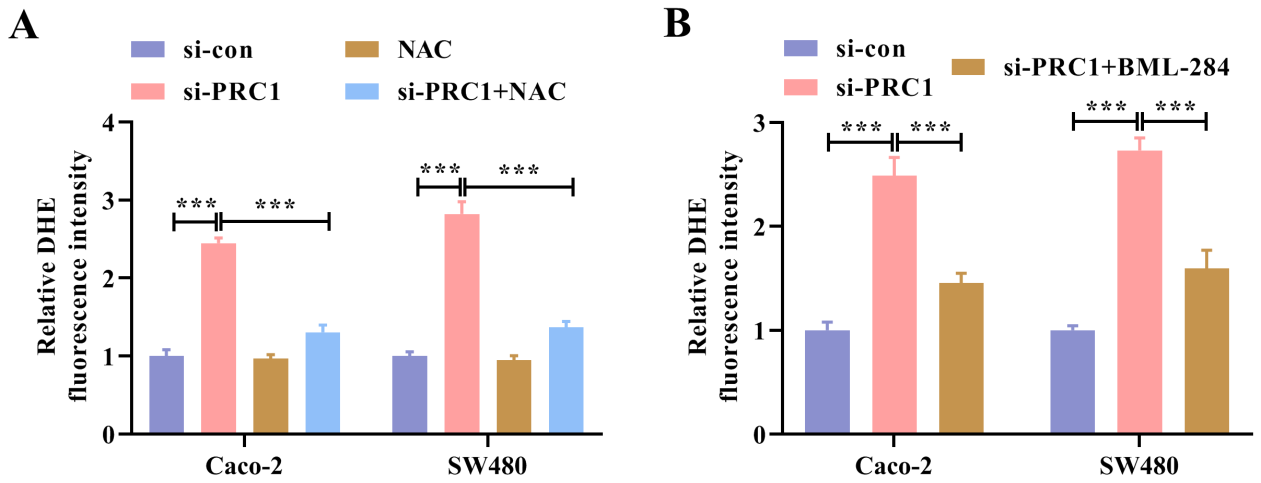


Fig. S2. Activation of Wnt/*β*-catenin by BML-284 could partially reverse the effects of PRC1 knockdown on ROS production in CRC cells. (A) CRC cells were pretreated with the ROS scavenger NAC (2 mM) for 2 h prior to gene transfection to block ROS generation. (B) CRC cells were transfected with si-PRC1 and then treated with 0.5 µM BML-284 (Wnt signaling activator) for 48 h. ^***^*P* < 0.001.


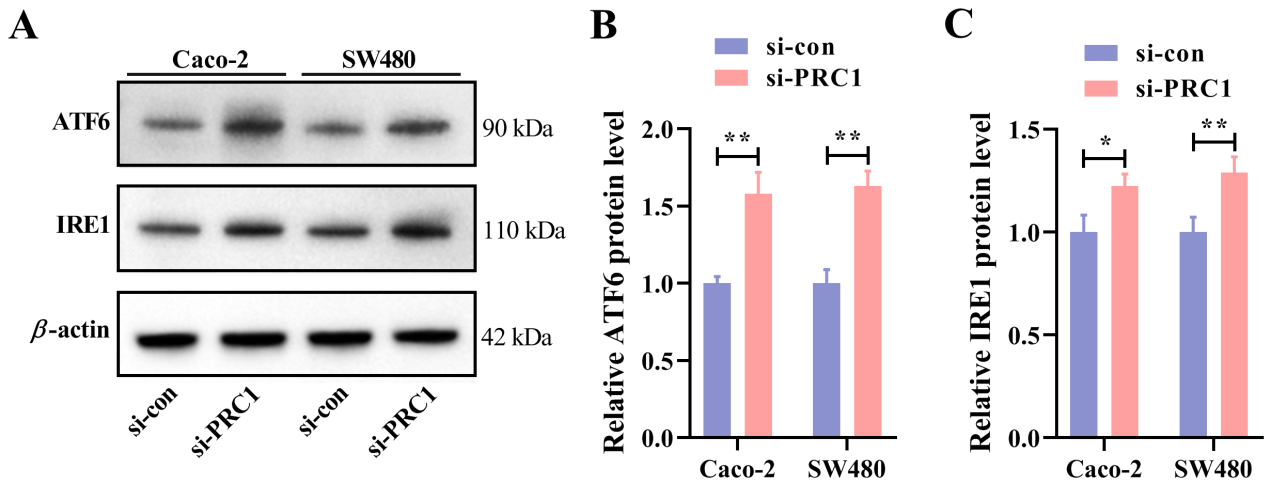


Fig. S3. PRC1 silencing upregulates the protein expression of ATF6 and IRE1 in CRC cells. Western blot analysis was performed to detect the protein levels of ER stress markers (ATF6 and IRE1) in CRC cells with PRC1 knockdown. Results showed that PRC1 knockdown resulted in elevated protein levels of ATF6 and IRE1 in CRC cells. ^*^*P* < 0.05 and ^**^*P* < 0.01.
